# Identify the Micro-Parameters for Optimized Discrete Element Models of Granular Materials in Two Dimensions Using Hexagonal Close-Packed Structures

**DOI:** 10.3390/ma16083073

**Published:** 2023-04-13

**Authors:** Xiaodong Zhou, Dongzhao Jin, Dongdong Ge, Siyu Chen, Zhanping You

**Affiliations:** 1 Rizhao City Transportation Bureau, Rizhao 276800, China; xzhou3@mtu.edu; 2 Department of Civil, Environmental, and Geospatial Engineering, Michigan Technological University, 1400 Townsend Drive, Houghton, MI 49931-1295, USAdge1@csust.edu.cn (D.G.); siychen@mtu.edu (S.C.); 3 National Engineering Research Center of Highway Maintenance Technology, Changsha University of Science & Technology, Changsha 410114, China; 4 School of Transportation, Southeast University, Nanjing 211189, China

**Keywords:** granular material, discrete element method, modeling theories, hexagonal close-packed structure, asphalt mixture

## Abstract

The widely used simple cubic-centered (SCC) model structure has limitations in handling diagonal loading and accurately representing Poisson’s ratio. Therefore, the objective of this study is to develop a set of modeling procedures for granular material discrete element models (DEM) with high efficiency, low cost, reliable accuracy, and wide application. The new modeling procedures use coarse aggregate templates from an aggregate database to improve simulation accuracy and use geometry information from the random generation method to create virtual specimens. The hexagonal close-packed (HCP) structure, which has advantages in simulating shear failure and Poisson’s ratio, was employed instead of the SCC structure. The corresponding mechanical calculation for contact micro-parameters was then derived and verified through simple stiffness/bond tests and complete indirect tensile (IDT) tests of a set of asphalt mixture specimens. The results showed that (1) a new set of modeling procedures using the hexagonal close-packed (HCP) structure was proposed and was proved to be effective, (2) micro-parameters of the DEM models were transit form material macro-parameters based on a set of equations that were derived based on basic configuration and mechanism of discrete element theories, and (3) that the results from IDT tests prove that the new approach to determining model micro-parameters based on mechanical calculation is reliable. This new approach may enable a wider and deeper application of the HCP structure DEM models in the research of granular material.

## 1. Introduction

The origin of the discrete element method (DEM) can be traced back to the late 1970s, when it was developed by Cundall and Strack to address the complexities associated with granular materials, owing to their inherently discrete nature [1]. Then, the DEM was introduced into the modeling of the asphalt mixture. Buttlar and You utilized it to model and examine the workings and efficiency of asphalt materials [2,3]. Another study simulated the viscoelastic behavior of asphalt mixture using micromechanical parameters obtained from a dynamic shear rheometer in the Simple Performance Test (SPT) [4]. A 3D microstructure-based Discrete Element Method (DEM) model was created by combining multiple 2D models and then was used to calculate the stress-strain behavior during repeated loading conditions [5]. The results of a laboratory test indicated that the 3D model had better agreement with the test results than the associated 2D models [6]. The contact models form the foundational mechanism in the DEM, and its micro-parameter determination is a critical part. Researchers developed several approaches to relate the micro-parameters with the macro-parameters of the asphalt mixture. For the compacted asphalt mixture at room temperature, the dynamic modulus test was used to determine the viscoelastic parameters of the Burgers model [7]. The dynamic modulus could be used to reflect the stress and strain response by specific load directly. The dynamic modulus test is conducted at temperatures of −10 °C, 10 °C, 21 °C, 37 °C, and 54 °C at loading frequencies of 0.1 Hz, 0.5 Hz, 1 Hz, 5 Hz, 10 Hz, and 25 Hz at each temperature and is specified in AASHTO T342. The simulation results were in agreement with the results obtained from laboratory tests. The creep test was used to determine the viscoelastic parameters in models that were based on microstructure [8]. The dynamic shear rheometer test [9] and the constant strain rate uniaxial compression test were conducted to calculate the time-dependent contact stiffness of the Burgers model [10,11]. Similar contact models and parameter calculations were used in the prediction of the mechanical properties of asphalt [12]. The internal forces configuration of the asphalt mixture was evaluated through the use of established DEM models. The parameter determination for the samples in the compaction process is more difficult than the compacted samples due to the high flowability of asphalt at high temperatures. Chen, Huang et al. proposed an indirect approach to predict the viscoelastic parameters at high temperatures [13]. In their study, serval dynamic modulus tests were performed at low temperatures, and the nonlinear regression analysis was used to obtain the mater curve of the asphalt mixture. Then, the viscoelastic parameters at high temperatures were predicted through the asphalt mixture master curve [14,15].

Another crucial aspect of the DEM model is the use of modeling techniques. You and Buttlar were pioneers in utilizing image processing to construct 2D model structures. They utilized grayscale images obtained through optical scanning to establish microfabric DEM models [2,3]. The simple performance test employed similar models to forecast the dynamic modulus and phase angles of the asphalt mixture [4]. The concept of constructing a 3D model by stacking 2D models was developed based on the 2D DEM models [5]. Then, the development of 3D DEM models utilizing ball/clump elements as basic building blocks was carried out. Ball elements are widely adopted in 3D DEM models for simulating the performance of asphalt mixture due to its simplicity and clear visual aid. For example, the uniaxial compression test was simulated by ball-based DEM models [10]. The modified model was demonstrated as having the capability of simulating creep tests. For the purpose of simplifying the DEM models for asphalt mixtures, researchers typically treat the mixture as a two-phase material composed of coarse aggregates and asphalt mastic [16]. The ball elements were also utilized to represent the asphalt mastic, which is composed of fine aggregate, fines, and asphalt. The ball-based models have an obvious disadvantage due to their inability to represent the irregular shapes of aggregates. New modeling approaches were developed to model the aggregates with more realistic shapes. A proposal was made to utilize randomly generated irregular particles to visualize and simulate the micro-scale properties of the asphalt mixture under mechanical loading [17]. The investigation into the effect of aggregate shape on the diffusivity of asphalt mastic utilized random packing models of ellipsoidal and convex polyhedral particles [18]. Additionally, researchers have utilized realistic aggregate shapes to improve the accuracy of asphalt mixture simulations. Techniques such as X-ray CT and image processing were employed to generate DEM models featuring realistic aggregate shapes [19,20]. A more precise method, the individual aggregate reconstruction technology, was proposed to establish DEM models for asphalt mixture [21,22]. Fracture behavior in asphalt concrete laboratory specimens is able to bridge a vital link in the design of asphalt concrete paving mixtures and pavement structures. A two-dimensional particle flow software package (PFC-2D) was used to study the complex crack behavior observed in asphalt concrete fracture tests [23]. A computer simulation using the discrete element method (DEM) is presented in order to understand and visualize how crushing initiates and develops inside a simulated pavement structure [24,25]. Yu et al. [26] studied the effect of aggregate size distribution and angularity distribution on dynamic modulus using a 3D discrete element method (DEM).

The above research primarily focused on the properties of the compacted asphalt mixture. The compaction process of asphalt mixture is characterized by frequent and intense material movement and changes in contact force. As a result, the contact models and modeling techniques for this process are distinct from those used for compacted asphalt mixtures. There are limited studies that focus on the compaction of asphalt mixture. Wang et al. compared the fundamental mechanics of asphalt compaction using both FEM and DEM and emphasized that DEM can simulate aggregate translation and rotation [27]. The DEM models have also been shown to provide valuable theoretical support for intelligent compaction. Chen and Huang et al. utilized the Burgers model to simulate the compaction of asphalt mixture using DEM [28]. In a subsequent study, they simulated gyratory compaction, vibration compaction, and kneading compaction using an open-source code [13]. Gong et al. established shape-based DEM models to simulate Superpave gyratory compactor (SGC) tests, which simulate the field compaction process of asphalt mixture, and reported agreement between the results of laboratory compaction tests and simulation results [29,30]. This research introduced realistic aggregate shapes into the DEM simulation. However, the established models had limitations on the total number of elements, compaction dynamics, and parameters determination.

Yu Liu et al. [7] introduced a set of theoretical calculations for DEM models using the Burgers contact model and cubic-centered cubic (SCC) ball array structures. This theoretical calculation approach has been proved reliable in predicting the dynamic modulus of asphalt mixture. In order to extend the use of DEM to a variety of performance tests, the hexagonal close-packed (HCP) ball array was used as the basic model structure. It is clear that the HCP structure is more complicated than the SCC structure and has a different mechanical structure. Therefore, a new set of theoretical calculations is needed.

This study aims to establish a hexagonal close-packed discrete element model for granular material with the ability to transit diagonal loading and performance based on Poisson’s ratio. To achieve this objective, first, new generation procedures of the HCP ball array for the 2D DEM models were proposed; second, a theatrical approach that was used to transition from material macro-properties to contact micro-parameters values in the 2D DEM models were derived based on the basic configuration and mechanism of discrete element theories; and third, the contact stiffness, bond strength, and an-isotropic properties were discussed and verified by comparing IDT results between designed 2D DEM models and laboratory tests.

## 2. Model and Methods

**Figure 1:**
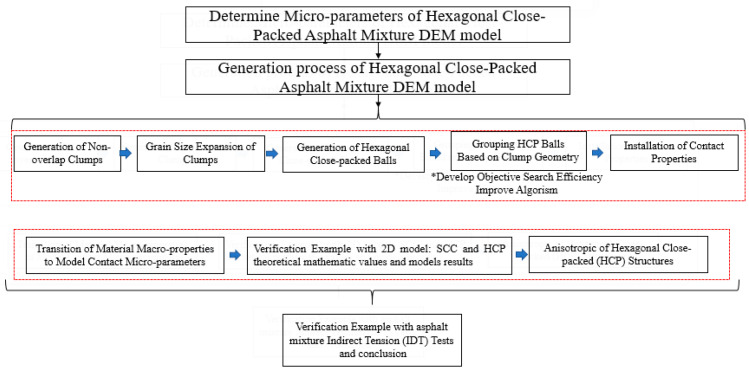
Research methodology in this research.

The research methodology of this study is shown in Figure 1, where the generation method of the new HCP model is introduced in the modeling procedures of the hexagonal close-packed generation method section. The process involves several steps, starting with the generation of nonoverlap clumps obtained through scanning aggregates of varying grain sizes with a 3D scanner, followed by grain-size expansion of clumps where the clump sizes are progressively increased to reach their intended dimensions. Next, hexagonal close-packed (HCP) balls are generated, and these balls are then grouped based on clump geometry, where the classification of an HCP ball as either coarse aggregate or asphalt mastic depends on whether its center position falls within a clump. Finally, the installation of contact properties is carried out using the contact-bond model, which has been shown to effectively simulate the fracture behavior of asphalt mixtures in previous studies. The corresponding mechanical calculation for contact micro-parameters was then derived and verified through simple stiffness/bond tests in the 2D DEM model and verified with theoretical values. Finally, an indirect tensile (IDT) test in the 3D DEM modeling generated by the HCP model structure and laboratory test results is compared.

**Figure 2:**
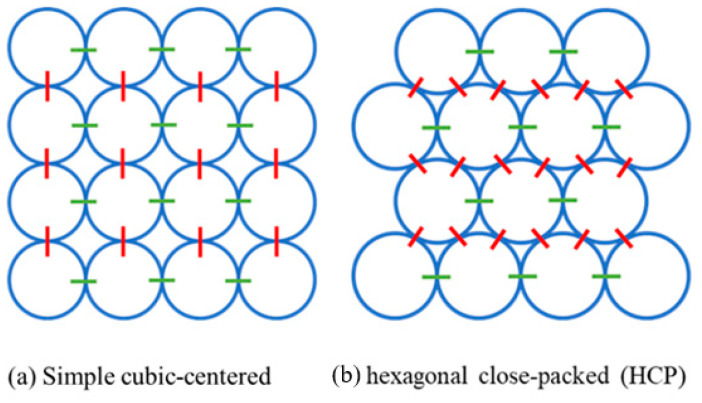
Two different types of the model structure used in DEM modelling (2D view).

In previous studies, the simple cubic-centered (SCC) ball array was widely used as the basic model structure, as shown in Figure 2a. This kind of model is proved to be effective in the simulation and in the prediction of the dynamic modulus of the asphalt mixture. The SCC structure can transit loading in vertical and horizontal directions efficiently. However, it lacks the ability to transit diagonal loading and performance based on Poisson’s ratio. To make up for this disadvantage, the hexagonal close-packed (HCP) ball array (see Figure 2b) was used.

This study aims to carry out a reliable approach to determine the micro-parameters in the DEM models. Based on the basic configuration and mechanism of discrete element theories, the transition from material macro-properties to contact micro-parameters was derived. The contact stiffness, bond strength, and an-isotropic properties were discussed and verified by designed DEM models.

### 2.1. Contact Stiffness (kn) without Bonding

#### 2.1.1. Case of SCC

**Figure 3:**
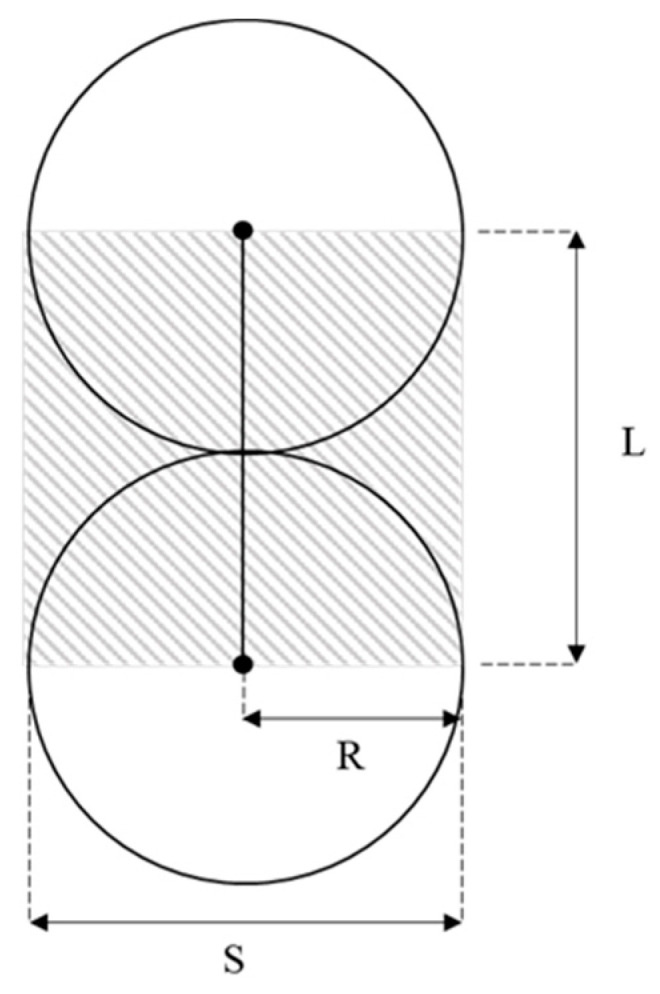
Basic Mechanical Unit of SCC Ball Array.

The basic mechanical unit of SCC can be described as a single contact with two balls (Figure 3). This unit can be treated as a single-spring system. The contact force (F) and stress (σ) can be expressed as Equations (1) and (2).

(1) $$F=k_{n}\;\cdot \;\delta$$

(2) σ=E · ε

where, $k_{n}$ is stiffness of the contact, δ is the displacement at the contact, E is the material modulus, and ε is strain.

In regard to unit dimensions, the stress and strain at contact can also be expressed as:

(3) $$\sigma =\frac{F}{S}\;$$

(4) $$\varepsilon =\frac{\delta }{L}\;$$

where S is the area of the contact plane and L is the length of contact.

Submit Equations (2)–(4) into Equation (1)

(5) $$E=\frac{L}{S}k_{n},\;L=2R,\;S=2R$$

where R is the radius of SCC balls. One important point to mention is that the third dimension L is hidden in the calculation of S.

Eventually, the material modulus (*E*) of an SCC array can be expressed by contact stiffness (*k_n_*):

(6) $$\begin{array}{c} E \end{array}=k_{n}$$

#### 2.1.2. Case of HCP

**Figure 4:**
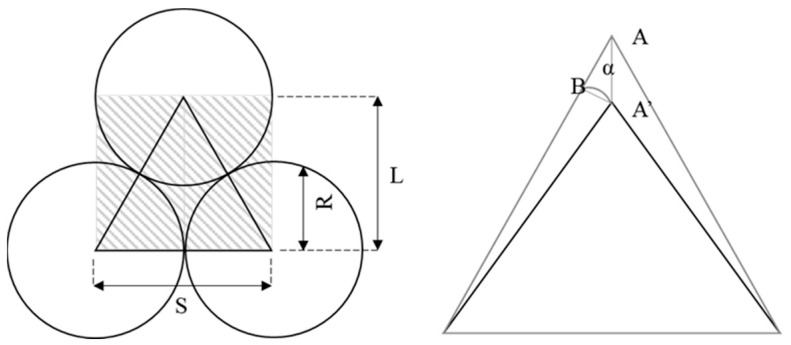
Basic Mechanical Unit of HCP Ball Array.

The basic unit of HCP is the combination of three closed contact balls, as seen in Figure 4. This unit can be treated as a simple truss system. The contact force F and displacement δ are the combinations of the vertical portion of $F^{\prime }$ and $\delta^{\prime }$:

(7) $$F=2\;\cdot \;F^{\prime }\;\cdot \;\cos\theta \;$$

(8) $$F^{\prime }=k_{n}\;\cdot \;\delta^{\prime }$$

(9) $$\delta^{\prime }=\delta \;\cdot \;\cos\theta$$

As the angle θ of the truss equals 30 degrees, the contact force F can be expressed as:

(10) $$F=2{cos}^{2}\theta \;\cdot \;k_{n}\;\cdot \;\delta =\frac{3}{2}k_{n}\;\cdot \;\delta \;$$

The length of the truss system is calculated as the vertical portion of the connection between the two balls:

(11) L=2cosθR, S=2R 

Eventually, the material modulus (*E*) of a unidimensional hex array can be related to contact stiffness (*k_n_*) as:

(12) $$E=\frac{3\sqrt{3}}{4}k_{n}\;$$

This section may be divided by subheadings. It should provide a concise and precise description of the experimental results and their interpretation as well as the experimental conclusions that can be drawn.

#### 2.1.3. Validation Example

**Figure 5:**
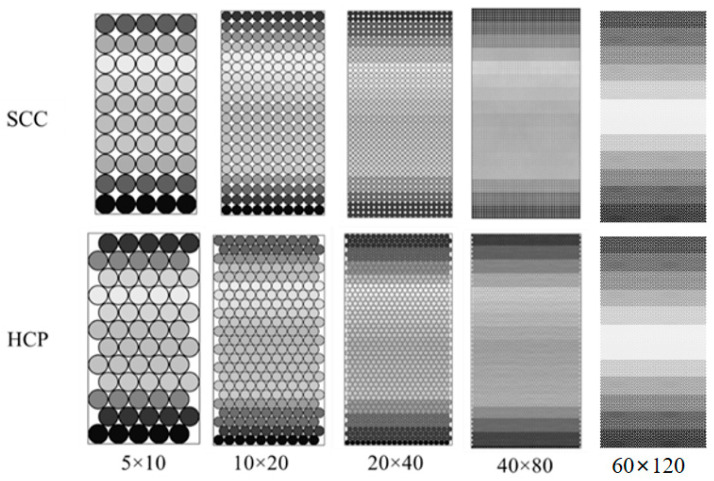
Stiffness Validation Models of SCC and HCP Ball Arrays.

A set of DEM models was used to verify the reliability of Equations (6) and (12). The ratio of model height versus model width was set as 2.0. Due to hardware and computational power limitations, the validation model dimension is constrained to a limited size, which is worth noting. Consider the impact of model sizes, as shown in Figure 5, where four groups of models were tested with scales ranging from 5 × 10, 10 × 20, and 20 × 40 to 40 × 80.

**Figure 6:**
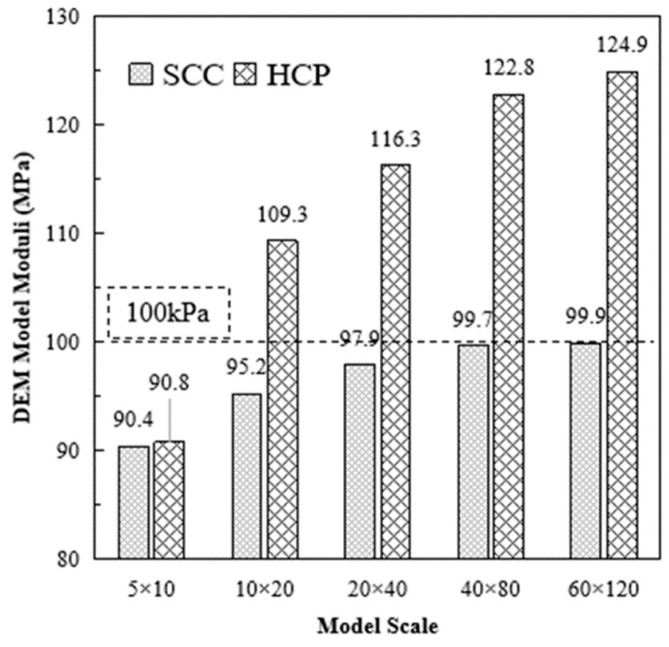
DEM Model Moduli of SCC and HCP Stiffness Validation Models without Boding.

The contact stiffness was set as 1 × 10^5^ for all the tested models. All boundaries were rigid and confined. Vertical displacements of 1% model height per second were applied on the top plane, according to Equations (6) and (22). The theoretical material moduli should be 100 kPa and 129.8 kPa. The obtained material moduli from DEM model are shown in Figure 6.

The model scale has a significant impact on the obtained material moduli from the DEM model. HCP was more sensitive to the model scale than was SCC. The obtained material moduli of the SCC group from the DEM model were close to the 100 kPa theoretical value. The HCP group reached 96.22% of the theoretical value (124.9/129.8 kPa) when using the 60 × 120 configuration.

### 2.2. Contact Stiffness (kn) with Bonding

#### 2.2.1. Case of SCC 

The bonding condition makes no difference to the SCC ball arrays, since the bonding plane is perpendicular to the vertical direction.

#### 2.2.2. Case of HCP

**Figure 7:**
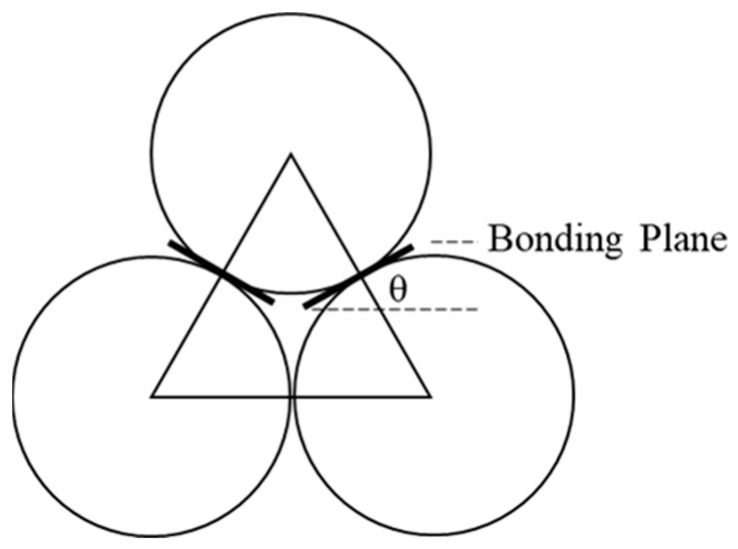
Bonding Plane in Basic Mechanical Unit of HCP Ball Array.

The bonding condition makes no difference to the SCC ball arrays, since the bonding plane is perpendicular to the vertical direction. The bonding plane in the HCP ball arrays has an angle of θ degrees in the horizontal direction (Figure 7). Thus, the shear force at the bonding plane contributes a vertical component when applied to vertical loading.

The shear force at contact plane can be expressed as:

(13) $$F_{s}=\left\{\begin{array}{c} k_{s}\delta_{s},\;\;before\;slip\\ \mu F_{n},\;\;slip \end{array}\right.$$

(14) $$\delta_{n}=\delta cos\theta ,\;\delta_{s}=\delta sin\theta$$

where $k_{s}$ is the shear stiffness at bonding plane, $\delta_{s}$ is the displacement in shear direction, μ is the friction coefficient, and $F_{n}$ is the normal contact force at bonding plane.

Then, the total force in vertical direction equals

(15) $$F=F_{n}cos\theta +F_{s}sin\theta$$

Submit Equations (13) and (14) into (15):

(16) $$F=2(k_{n}cos^{2}\theta +k_{s}sin^{2}\theta )\delta$$

The material modulus before slip then equals

(17) $$E=\frac{\frac{F}{S}}{\frac{\delta }{L}}=\frac{2(k_{n}cos^{2}\theta +k_{s}sin^{2}\theta )\delta /2R}{\frac{\delta }{2Rcos\theta }}=\frac{3\sqrt{3}}{4}k_{n}+\frac{\sqrt{3}}{4}k_{s}\;$$

#### 2.2.3. Case of HCP

**Figure 8:**
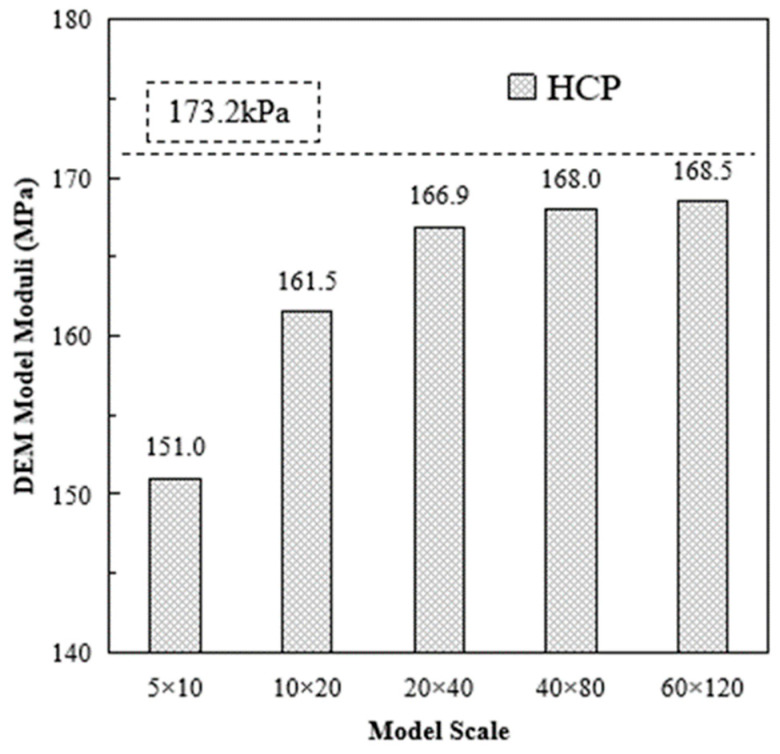
Obtained Moduli of HCP Stiffness validation Models with Bonding.

The configuration of the validation example is the same as the models used in the previous section. Contact stiffness (*k_n_*) without bonding $k_{n}$ and $k_{s}$ were set as 1 × 10^5^. The theoretical value according to Equation (17) was 173.2 kPa. The simulation results are shown in Figure 8.

Model scale also has significant impacts on the material moduli of DEM models. As the model scale increased, the obtained material moduli from the DEM model were closer to the theoretical value. When using the 60 × 120 configuration, the obtained material moduli from the DEM model reached 97.29% (168.5/173.2) of the theoretical value. Considering the hardware calculation efficiency and the scale effects influence, a 40 × 80 configuration was used in this study.

### 2.3. Contact Bond Strength

#### 2.3.1. Case of SCC

The tensile bond strength $T_{F}$ can be expressed as:

(18) $$\begin{array}{c} T_{F}=T_{\sigma }\;\cdot \;S \end{array}$$

In the case of the unidimensional ball array, refer to Equation (5):

(19) $$T_{\sigma }=\frac{T_{F}}{2Rt},\;t=1\;$$

#### 2.3.2. Case of HCP

The tensile bond strength $T_{F}$ can be expressed as:

(20) $$T_{F}=2T_{\sigma }cos\theta =\sqrt{3}T_{\sigma }$$

(21) $$T_{\sigma }=\frac{\sqrt{3}T_{F}}{2Rt},\;t=1$$

The relationship of contact stiffness in the contact interface is:

(22) $$\frac{1}{k_{n}}=\frac{1}{k_{n1}}+\frac{1}{k_{n2}}.$$

where *k_n_* is the aggregate-mastic interface normal contact stiffness, *k_n_*_1_ is the normal contact stiffness of aggregate, and *k_n_*_2_ is the normal contact stiffness of mastic; *k_s_* used the same method.

#### 2.3.3. Validation Example

Setting $T_{F}=0.5\;N$ and R=0.01 m, according to Equations (19) and (21), the theoretical values of SCC and HCP models are 25 Pa and 43.3 Pa, respectively. The obtained value from DEM model are 24.7 Pa (98.8% of theoretical value) and 38.37 Pa (88.6% of theoretical value), respectively.

### 2.4. An-Isotropic of Hexagonal Close-Packed (HCP) Structures

The vertical direction and horizontal direction of the HCP ball array are different. The an-isotropic properties can be written as:

$$E=\frac{sin\varphi }{sin\theta }\left|i\right|k_{n}+\left(cos\varphi -\frac{sin\varphi }{sin\theta }\cos\theta \right)\left|j\right|k_{s}$$

in which, *ϕ* is among 0–30 degree, *θ* equals to 30 degrees.

**Figure 9:**
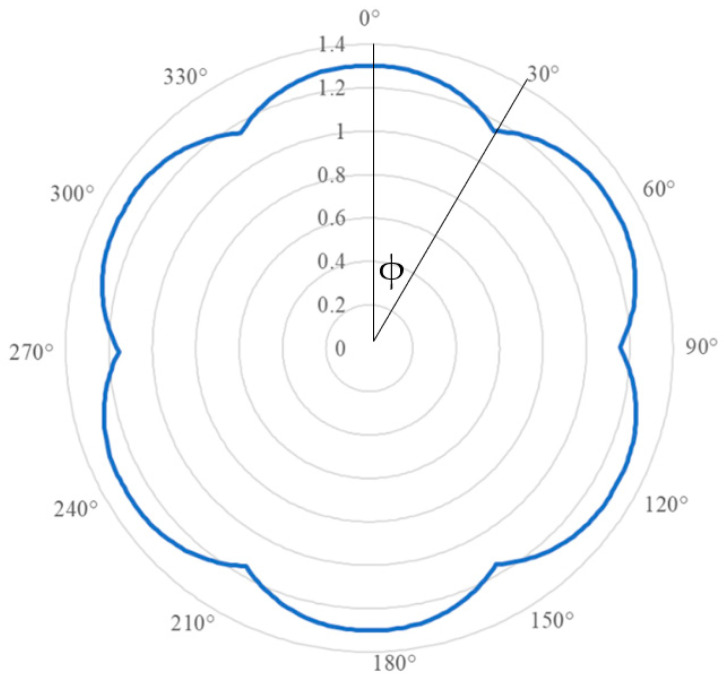
Material Moduli An-isotropic of Hexagonal Close-Packed (HCP) Structures.

In the case of material modulus, the modulus constant in the horizontal direction is $\left|i\right|=\frac{2\sqrt{3}}{3}$, and in the vertical direction it is $\left|j\right|=\frac{3\sqrt{3}}{4}$. Then, the an-isotropic properties of the material are plotted in Figure 9.

## 3. Modeling Procedures of Modified Random Generation Method with Realistic Coarse Aggregate Shapes

To enhance the efficiency of the model, a modified random generation method with realistic coarse aggregate shapes was introduced in this study. The new method employed the cross-section of 3D models as the 2D model geometries rather than the directed generation of 2D models. The new modeling procedures are described in the following steps:

- Generation of Nonoverlap Clumps

**Figure 10:**
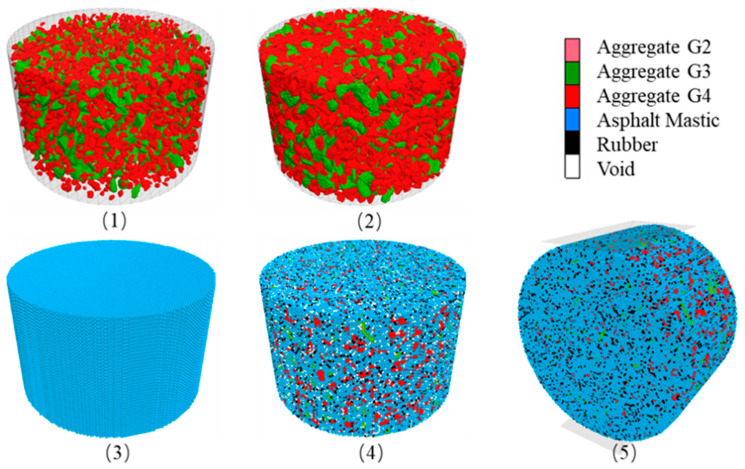
Model setup process of the rubber modified HMA: (**1**) clumps generation process; (**2**) diameter expansion procedure of clumps; (**3**) HCP balls generation process; (**4**) setup of different group of HCP balls; (**5**) indirect tensile test process.

The clump geometries were obtained through scanning aggregates of varying grain sizes using a 3D scanner. The methods for generating clumps and determining grain size have been described in prior studies [21,31]. The clump grain sizes were determined based on the mixture design. The clumps were generated within a 100 × 63 mm cylinder container, with each clump being generated at 70% of its intended grain size to ensure successful generation. The coarse aggregates of grain sizes G2, G3, and G4 were generated in succession, as depicted in Figure 10(1). It is worth mentioning that the coarse aggregate can also be directly introduced via a compacted model through the compaction process.

- Grain-Size Expansion of Clumps

The clump grain sizes were increased until they reached their target dimensions, as depicted in Figure 10(2). The expansion procedure involved several iterations to prevent excessive overlapping in a single expansion. In the example shown, the clump grain sizes were expanded 10 times with an expansion factor of approximately 1.03631121 for each step, calculated as (1/0.7) (1/10). To minimize overlap between clumps, the model was run until the maximum overlap ratio dropped below 0.1%. While the overlap ratio could be calculated by iterating through the entire clump set, this method would add unnecessary computational strain to the computer. As an alternative, the maximum overlap ratio could be estimated by monitoring the leading contact force. To limit clump movement and enhance efficiency, a high damping ratio of 0.7 was assigned to all clumps.

- Generation of Hexagonal Close-Packed (HCP) Balls

The two lattice structures that result in the highest density for equal-diameter ball arrangements are the cubic-centered cubic (SCC) and the hexagonal close-packed (HCP). In this study, the hexagonal arrangement was chosen. For ease of ball labeling, the balls were generated within a cubic space, and then any balls outside the cylindrical container boundary were removed, as illustrated in Figure 10(3).

- Grouping HCP Balls Based on Clump Geometry (Objective Search Efficiency Improved Algorism)

**Figure 11:**
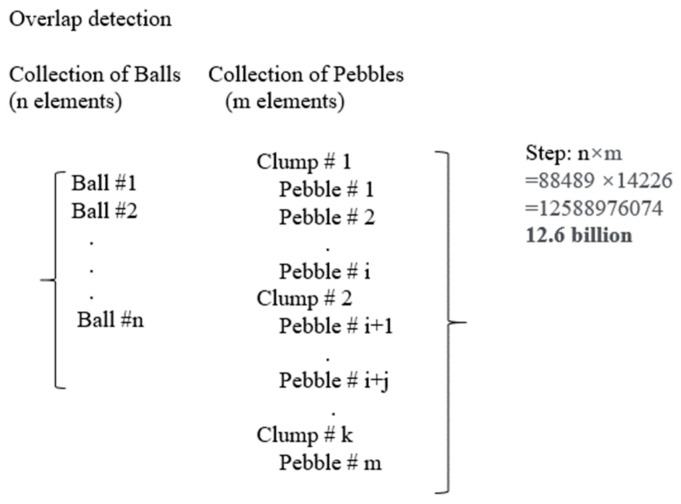
Original Objective Search Algorism.

The classification of an HCP ball into either coarse aggregate or asphalt mastic depends on whether its center position falls within a clump. The number of HCP balls representing rubber particles and voids was determined based on the mixture design. These two groups of HCP balls were then randomly selected from within the mastic. The final grouping results are displayed in Figure 10(4). The most time-consuming step in this section is the objective search of overlap detection, which determines the group properties of HCP balls. Thus, here we proposed an improved objective algorism. The original algorism needs to loop the ball list and clump list (pebble list) from beginning to end, as shown in Figure 11. The required steps for a model with 88,489 balls and 142,266 pebbles are 12.6 billion.

**Figure 12:**
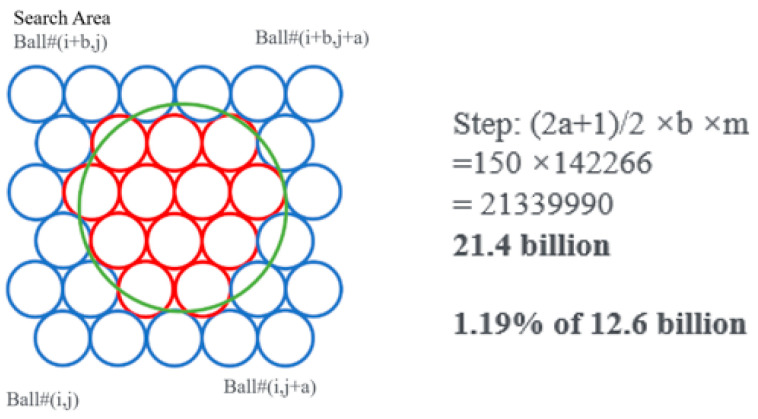
Illustration of the Objective Search Algorism: Clump pebble (Green Circle), Search Area (Blue Circle), Aggregate Cluster (Red Circle).

The improved algorism decreases the calculation steps by narrowing down the search area, as shown in Figure 12.

- First, find the location and diameter of the current pebble.
- Second, calculate the extended coverage area.
- Third, determine if the ball is within the pebble area.

By estimation, the improved objective algorism requires 21.4 million steps to finish the calculation, which saves about 98.9% of calculation time.

- Installation of Contact Properties

The contact-bond model was selected because it has been demonstrated to effectively simulate the fracture behavior of asphalt mixtures. Although nearly all aggregates and rubber particles are covered by asphalt, there is bond strength between directly connected aggregates and rubber particles. Furthermore, the linear contact model was designated as the default model for all subsequent contacts (following fracture), and the contact properties would be derived from the parent particles.

## 4. Validation Example with Indirect Tension (IDT) Tests

The IDT test is an effective method for evaluating the low-temperature cracking performance of asphalt mixture [32]. This section designed a group of indirect tension (IDT) tests in laboratory to verify the reliability of the proposed mechanical parameters transition.

### 4.1. Mixture Design and DEM models

**Figure 13:**
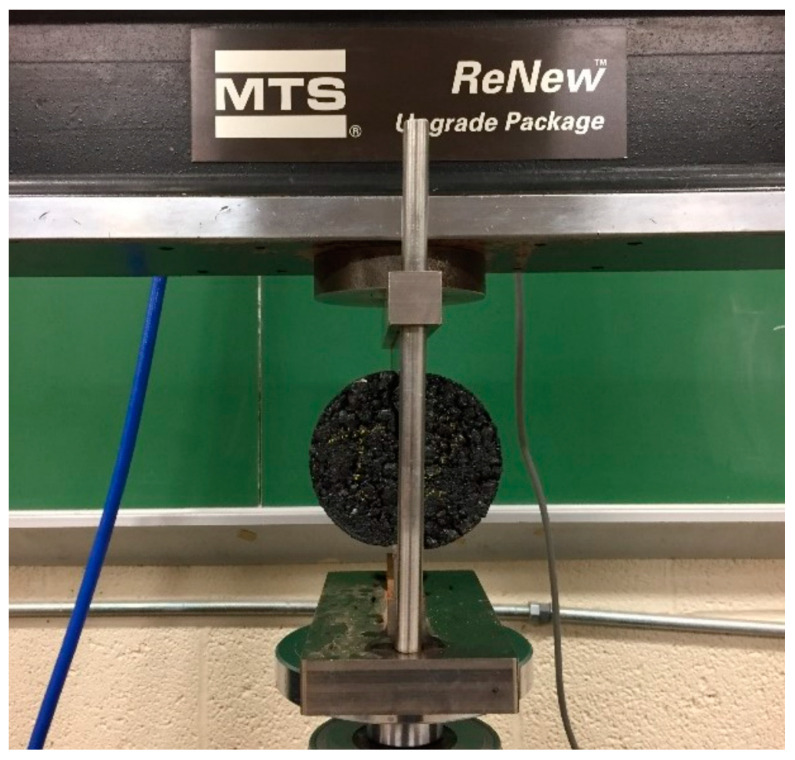
Indirect tensile strength test of asphalt mixture.

Three mixture designs were selected; see Table 1. The IDT test setup is shown in Figure 13. The test speed is 50 mm/s, and the load and displacements during the test are recorded and compared with the DEM model.

**Figure 14:**
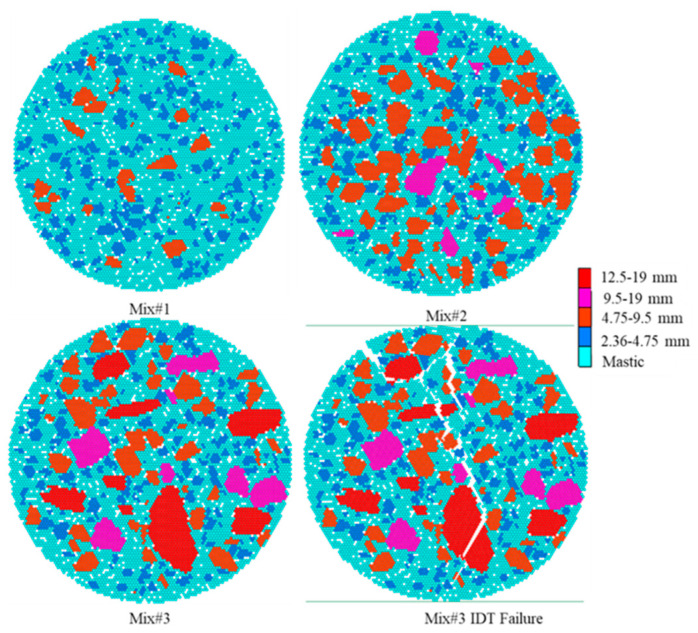
DEM Models for Parameter Validation Tests.

The models with three mixture designs are shown in Figure 14.

### 4.2. Calculation of Model Micro-Parameters

The micro-parameters were calculated based on the equations derived from the previous section, and the contact model parameters were calculated based on materials’ macro-properties [7,33,34,35], as shown in Table 2.

### 4.3. IDT Results and Discussion

**Figure 15:**
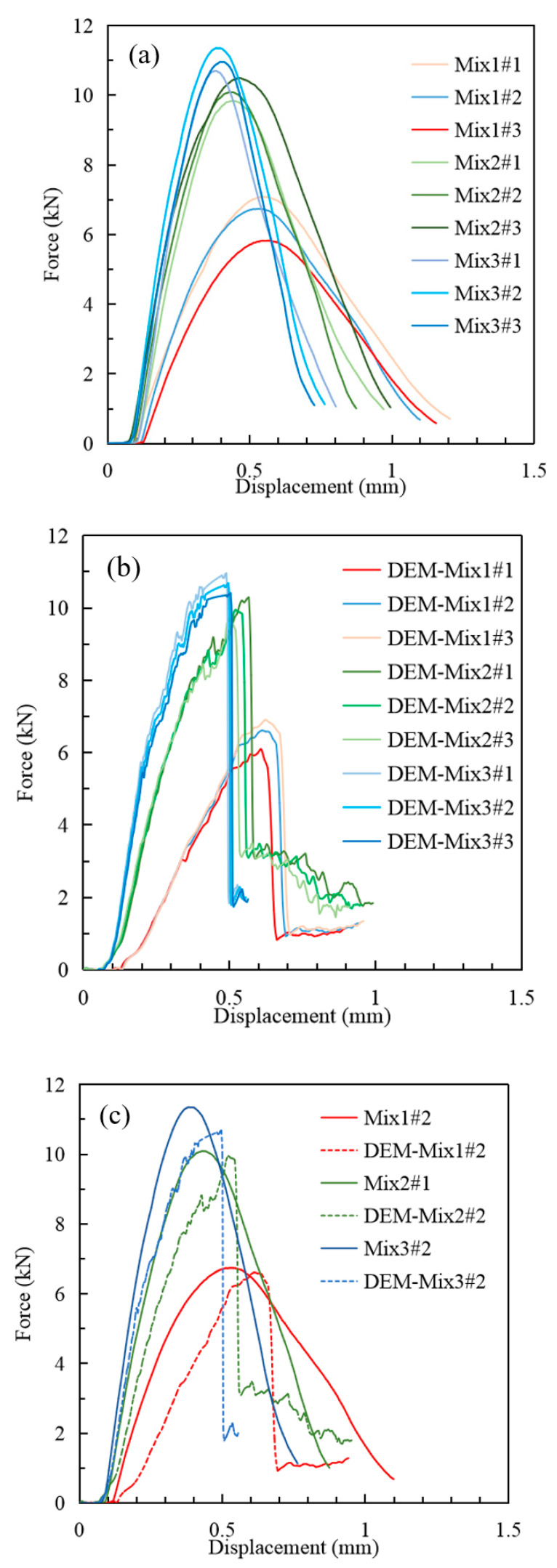
IDT Results from Laboratory Tests and DEM simulation. (**a**) Laboratory Tests. (**b**) DEM Simulation. (**c**) Comparison between Laboratory Tests and DEM Simulation.

The IDT results from laboratory tests and DEM simulation are shown in Figure 15. As shown in Figure 15a, a total of 9 laboratory specimens belonging to 3 groups were tested. For Mix1, Mix2, and Mix3, the average peak forces were 6.25 kN, 10.12 kN, and 10.81 kN, respectively. In general, the mixture type has the largest coarse aggregate grain size (Mxi3) and presented the highest peak force (tensile strength). Accordingly, Mix3 showed the steepest increasing rate (material moduli) and minimum displacement at the force peak (ultimate strain). After specimen failure, the decrease curves were relatively gentle compared to the increase curve. In this test, the standard deviations caused by the differences in specimens and test errors were relatively large but were still in a reasonable range.

Figure 15b shows the results of the DEM simulation for Mix1, Mix2, and Mix3. The average peak forces were 6.21 kN, 9.96 kN, and 10.56 kN, respectively. The results were close to that of laboratory tests, with relative errors ranging from 0.64% to 2.31%. The standard deviations of IDT results were at the same level as the comparison group. With zigzag data point curves, the results were much “rougher” than that of the laboratory control group. This is caused by the limited model scale. Actually, in the authors’ other studies there are models with millions of elements (less than 10,000 elements were used in this study) that could present more smooth curves, especially in 2D. The authors’ intention is to showcase the ability and reliability of their models by utilizing limited scales. After specimen failure, sharp drops were observed. There were two major reasons. First, the limited model scale led to large jumps at each of the failures, and second, the 2D models had less freedom than reality in which cracks could develop in lateral directions.

To compare the results, one force/displacement curve (whose test value is in the middle) for each mixture type was chosen, as shown in Figure 15c. The peak value of DEM simulations causes more displacement than the in lab because the initial loading stage of the DEM needs a process to “compact” the model into a denser status to achieve better loading transfer efficiency. However, prior to specimen failure, the peaking value and other parts of loading curves have good consistency with lab results. Considering that all the parameters are based on theoretical calculation without adjustment and with limitations on minimum element size, the DEM simulation results are reasonable, and the parameter calculation is reliable.

Compared to other models that utilize the discrete element method, which often exhibit a relative error exceeding 10%, the error in the results of this study is relatively small. It is noteworthy that the parameters in this study are derived using formulas rather than iteratively fitting them based on simulation results, as is commonly practiced in general studies. Additionally, this study employs a minimum-cost two-dimensional model with limited dimensions and scale, and the calculation of contact parameters is based on laboratory experiments that may have some degree of error fluctuation. Given these constraints, simulating loading curves of three different graded mixtures with consistent trends is still a challenging task, despite slight differences in the curves.

The crux of this paper lies in utilizing a theoretical calculation method to derive contact parameters for the discrete element method when simulating particulate matter instead of relying on iterative back-calculation fitting, as in typical research. Starting from a theoretical level, this method is more logical, resource-efficient, and reproducible.

## 5. Conclusions

In this study, a new approach for modeling procedures and determining parameters was proposed to enhance the integration of discrete element models (DEMs) in asphalt simulation. The following conclusions were drawn:

(1) A new approach for modeling procedures using the hexagonal close-packed (HCP) structure was proposed. This method, which employs realistic coarse aggregate morphology from 3D scanning, was demonstrated to be effective and can help save time and resources by reducing the need for laboratory samples. An objective search-efficiency improvement algorism is developed in this process.
(2) Micro-parameters of the DEM models were transformed from material macro-parameters using a set of equations that were derived based on the basic configuration and mechanism of discrete element theories. The effectiveness of the DEM models in simulating the indirect tensile strength for asphalt mixtures was demonstrated. The results were close to that of laboratory tests, with relative errors ranging from 0.64% to 2.31%.
(3) The key contribution of this research is the use of a reliable approach for determining model micro-parameters through mechanical calculation instead of a radical and inefficient iteration method for model parameter fitting. This new approach has the potential to expand and deepen the application of HCP structure DEM models in granular material research.

## Figures and Tables

**Table 1:** Mixture Design for Parameter Validation Tests.

Sieve Size (mm)	Passing (%)		
	Mix#1	Mix#2	Mix#3
19	100	100	100
12.5	100	100	94
9.5	100	97	86
4.75	94	75	71
2.36	69	54	54
1.18	46	36	38
0.6	32	25	26
0.3	20	15	16
0.15	13	7	8
0.075	8.5	4.8	4.4
Asphalt content (%)	7	5.8	5.4

**Table 2:** Martial Properties and Micro-parameter.

		Aggregate	Mastic	Aggregate-Mastic Interface
Elastic moduli, E, Pa	$E=\frac{3\sqrt{3}}{4}k_{n}+\frac{\sqrt{3}}{4}k_{s}$	20 GPa	300 MPa	-
Poisson’s ratio	$v=\frac{E}{2G}-1$	0.2	0.5	-
Tensile strength, σ, Pa	$\sigma =\frac{\sqrt{3}}{2}\frac{T_{f}}{R}\;$	15.27 MPa	7.04 MPa	6.33 MPa
Shear strength, τ, Pa	$\tau =\frac{2S_{f}}{R}$	30.54 MPa	13.45 MPa	12.10 MPa
Stiffness ratio,$\;k^{*}$	$k^{*}=\frac{k_{n}}{k_{s}}=2\left(v+1\right)$	2.4	3.0	2.33
Normal stiffness, $k_{n},\;N/m$	$k_{n}=\frac{4\sqrt{3}E}{3\left(3+k^{*}\right)}t$	7.23 × 10^8^	7.11 × 10^6^	1.41 × 10^7^
Shear stiffness, $k_{s},\;N/m$	$k_{s}=\frac{k_{n}}{k^{*}}$	3.01 × 10^8^	3.05 × 10^6^	6.04 × 10^6^
Tensile bond break force,$\;T_{f},\;N$	$T_{f}=\frac{2\sqrt{3}R\sigma }{3}t$	529.04	243.82	219.44
Shear bond break force, $S_{f},\;N$	$S_{f}=\frac{R\tau }{2}t$	458.16	201.69	181.52
Friction coefficient		1.07	0.58	0.58

## Data Availability

The datasets generated during analyzed during the current study are available from the corresponding author on reasonable request.
